# Natural and anthropogenic factors controlling hydrogeochemical processes in a fractured granite bedrock aquifer, Korea

**DOI:** 10.1007/s10661-025-14037-y

**Published:** 2025-04-30

**Authors:** Jiyun Kim, Jaeyeon Kim, Dugin Kaown, Won-Tak Joun

**Affiliations:** 1 School of Earth and Environmental Sciences, Seoul National University, Seoul, 08826 Republic of Korea; 2 Disposal Performance Demonstration R&D Division, Korea Atomic Energy Research Institute, Daejeon, 34057 Republic of Korea

**Keywords:** Hydrogeochemistry, Groundwater statistics, Radon, Stable isotopes, Conceptual models

## Abstract

Contamination of groundwater has become a critical environmental concern, prompting international inquiries. In this study, the impacts of natural and anthropogenic factors in the granite bedrock groundwater system were identified based on the hydrogeochemical compositions including environmental isotopes (δ^18^O, δ^2^H, ^222^Rn, δ^34^S_SO4_, δ^18^O_SO4_) using multivariate statistical methods. Hierarchical clustering analysis classified the groundwater samples into three groups for both dry and wet seasons. The first group, observed in both seasons, represents groundwater influenced by water–rock interactions in low flow and also demonstrates anthropogenic contamination near densely populated residential areas. The second group corresponds to higher flow groundwater, where surface water interaction affects with minimal anthropogenic impact. The third group characterizes relatively radon-contaminated groundwater, representing the predominant groundwater type in the study area. The isotope mixing model based on δ^34^S_SO4_ and δ^18^O_SO4_ identified proportional contributions of precipitation (~ 14%), sewage (~ 22%), soil (~ 78%), and sulfide oxidation (~ 27%) sources. The redox processes of bacterial sulfate reduction and sulfide oxidation were determined to have a minimal influence on sulfur isotope fractionation within the system. By integrating hydrogeochemical analysis, sulfur isotopes, and the MixSIAR model to trace sulfate sources, uncertainties are able be accounted in source contributions. The groundwater system was mainly influenced by natural factors through infiltration, particularly via the unsaturated soil layer during the wet season. This also indicates enhanced mixing of multiple factors during the recharge or discharge processes triggered by rainfall events. In contrast, anthropogenic contributions declined indicating strong seasonal influences, especially from sewage which decreased from 22 to 6% in groundwater most affected by human activity. This highlights the role of rainfall in diluting human-induced contaminants from the groundwater system. To understand the fractured granite groundwater system, a conceptual model was developed, detailing groundwater types and identifying sulfur sources.

**Supplementary Information:**

The online version contains supplementary material available at 10.1007/s10661-025-14037-y.

## Introduction

Groundwater is an interactive element of the hydrological cycle and is commonly acknowledged as an important resource for drinking water, irrigation, and numerous other uses. The contamination of groundwater has emerged as a pressing environmental issue. This leads to numerous global investigations aimed at identifying chemical trends in groundwater quality and assessing potential impacts on environmental components (Raimi et al., 2022; Ravindiran et al., 2023). Groundwater is susceptible to contamination from both natural and human-induced factors, worsening its quality due to growing human needs. Anthropogenic influences can be relatively easily controlled, whereas natural influences are more difficult to identify. Therefore, it is important to understand the hydrogeochemical attributes and contamination of groundwater. It is particularly essential when caused by natural factors, to ensure management of groundwater resources (Bondu et al., 2016; Kim & Lee, 2023; Wei et al., 2024).

Environmental stable isotopes, recognized as distinctive markers in the hydrological cycle, have acquired significant attention in water studies, especially the isotopes of water compositions (Liu et al., 2023a; Lyons et al., 2023; Ren et al., 2024). Sulfur isotopes in aquatic settings undergo noticeable natural fluctuations, leading to distinct isotopic patterns in sulfate originating from various sources unless the fractionation is affected by the flow path of the groundwater. Sulfate found in surface and groundwater can originate from diverse sources, including natural and human activities. Increased levels of sulfate are commonly indicative of the pollution through natural processes (Mao et al., 2023; Chen et al., 2024b; Qu et al., 2024; Wang et al., 2024; Zhang et al., 2024). Bacterial sulfate reduction is absent under oxidizing conditions. In this condition, the exchange of oxygen and sulfur isotopes in sulfate in groundwater is less affected by the isotope fractionation around the neutral pH range (Jakóbczyk-Karpierz & Ślósarczyk, 2022; Lloyd, 1967). This method has demonstrated effectiveness in identifying both natural and human-induced contributions in groundwater and surface water which may indicate insights into reaction pathways (Cao et al., 2018; Kaown et al., 2009; Ye et al., 2024; Zhang et al., 2015).

Daejeon is one of the metropolitan cities in South Korea, characterized by its predominant geological composition of granite, which covers more than 50% of the country (Cho et al., 2015; Yun et al., 2016, 2017). Residents in Daejeon commonly rely on groundwater for various purposes, yet the social concern of understanding the factors that influence the groundwater system within the granite aquifer remains challenging. As hard rock groundwater plays a vital role in supplying water resources in many regions, natural tracers have been employed to investigate the granite bedrock groundwater system using to reveal hydrogeochemical processes (Prasun & Singh, 2024; Chakan et al., 2024; Saka et al., 2024). Groundwater aquifers in granite terrains often contain high levels of natural contaminants such as radon and fluoride, but their distribution can vary within the same geological formation (Kim & Jeong, 2005; NIER & KIGAM, 2010; Yun et al., 2016; Hwang & Moon, 2021). The diversity in structure suggests that understanding the flow dynamics can be extremely intricate and difficult to confine. Granite bedrock is crystalline, and groundwater generally flows through fractures. Depending on the frequency, density, and other characteristics of these fractures, spatial heterogeneity can be induced. Furthermore, granite consists of biotite, pyrite, and uranium deposits. As a result, various chemical components are detected, leading to differing hydrochemical characteristics depending on factors such as groundwater flow paths and interactions with rock formations (Bochet et al., 2020; Roques et al., 2014; Smellie et al., 1995). The climate characteristic of distinct dry and wet seasons in South Korea induces unconventional hydraulic characteristics and alterations in flow direction or mixing processes (Kim & Lee, 2023; Taylor et al., 2013). Groundwater undergoes chemical changes through interactions with minerals in the aquifer or through mixing with other groundwater along its flow paths beneath the surface (Kumar et al., 2006; Toth, 1984). Therefore, investigating the hydrogeochemistry of this region becomes a foremost concern for society.

Although previous studies have investigated the origins and chemical evolution of granite groundwater using hydrogeochemical components including sulfur isotopes, a comprehensive integration of sulfur isotopes with radon and fluoride remains lacking. Such integration is essential to fully distinguish between natural and anthropogenic influences in granite bedrock aquifers. Further hydrogeochemical insights can be obtained through examining seasonal variations and evaluating extensive groundwater mixing and the hydraulic conditions created by fractured crystalline bedrock. This study aims to (1) reveal the differentiated hydrogeochemical characteristics of groundwater, (2) investigate seasonal variations by analyzing hydrogeochemical differences between dry and wet seasons, (3) identify natural and anthropogenic sulfur sources within the groundwater system and quantify their contributions, and (4) develop a conceptual model to enhance the understanding of hydrogeochemical processes influenced by environmental factors, groundwater flow, and aquifer properties within the fractured granite bedrock aquifer. This study contributes to integrated groundwater quality management by identifying sources of contamination, assessing climatic impacts, and understanding aquifer vulnerability.

## Materials and methods

### Study area description

**Fig. 1:**
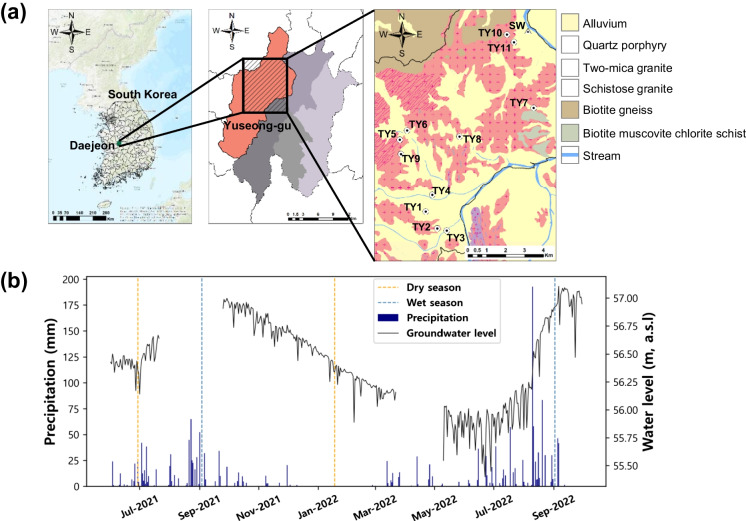
**a** Geological map of Yuseong-gu, Daejeon, South Korea, displaying the spatial distribution of lithology, stream, and sampling points including groundwater supply facilities (TY) and surface water (SW); **b** time series of Yuseong-gu precipitation and groundwater level of national monitoring well located throughout the sampling periods (2021-Jun- 01 ~ 2022-Sept- 30)

The study area was conducted at groundwater supply facilities of Yuseong-gu, Daejeon, located in the central part of South Korea Peninsula (Fig. 1a). The sampling points were selected to represent groundwater influenced by granite bedrock, which is common in the region. Most of the sites are located in residential and urban areas of Yuseong-gu, Daejeon. The investigation of potable and domestic groundwater sources from supply facilities in the study may provide practical insights for the management and evaluation of drinking water resources. The region of the area is adjacent to the main stream of Geum river basin in north to northeast of the area which is one of the five national rivers. Kapcheon stream is the largest branch stream of Geum river in Daejeon and is flowing south to northeast direction of Yuseong-gu district and consists the eastern part of the borderline.

The geology of the study area mostly consists of an alluvium layer and granite rocks. The alluvium layer was widely distributed, penetrating or enveloping the Jurassic granite rock, particularly close to the riverside of Gapcheon stream, consisting of unconsolidated sediments such as silt, sand, and gravel (Kim et al., 2020). Jurassic granite includes rock facies of Schistose granite and Two-mica granite, which surround the southern of Daejeon area. Calcite is known to exist by sealing fractures within the Jurassic granite rock. The main constituents of Schistose granite are quartz, orthoclase, microcline, oligoclase, biotite, and muscovite. The Two-mica granite is consisted with coarse grains of quartz, feldspars, and micas of muscovite and biotite. Quartz porphyry was observed intruding the Jurassic granitoids from south to north, and biotite-bearing gneiss and schists were also widely intruding the area. The Jurassic granite was locally altered along fractures, with chlorite and epidote forming alongside quartz veins in the hydrothermally altered zone. The fracture zone within the Jurassic granite exhibits diverse hydrothermal alterations, including sericitization, epidotization, and chloritization. Hydrothermally altered zones containing quartz or chlorite–epidote veinlets were found within the fractures or associated with alteration minerals such as pink feldspar, muscovite, calcite, and fluorite. Additionally, small amounts of sulfides were occasionally present in the hydrothermally altered zones of the granite (Park et al., 1977; Jeong, 2001; Moon et al., 2013; Ryu et al., 2012; Hwang et al., 2014; Cho & Choo, 2019; Hwang et al., 2023).

The common climate characteristic of South Korea is the East Asian monsoon, and the Kapcheon basin area is also affected by this climate. It experiences severe seasonal variation between a clear, pre-monsoon season and summer monsoon season primarily from July to August. This period is accompanied by frequent heavy rainfall event and storms (Lee et al., 1999). The mean monthly air temperature of Yuseong-gu district ranged between − 1.0 °C in January and 27.8 °C in July throughout 2021 and 2022. The annual precipitation of the study area in 2021 has recorded 1109.5 mm and 1203.1 mm in 2022. Approximately 60% of rain throughout 2021 and 2022 during the summer wet season with the extreme storm event in August, 2022 particularly contributed to the amount of precipitation of the season (Korea Meteorological Administration, http://www.kma.go.kr). The groundwater level of Sin-seong national groundwater monitoring well, located near the sampling points, was measured by the groundwater monitoring center in Korea.

The groundwater level was plotted with Yuseong-gu daily precipitation in Fig. 1b (National Groundwater Information Management Service, http://www.gims.go.kr). Even though part of data was missing during the measurement, groundwater level patterns in the study area were observed to fluctuate corresponding with the precipitation data. Heavy rainfall in August, 2022 (192.90 mm) recorded almost three times higher than the maximum precipitation recorded in 2021 (65.00 mm). Along with frequent rain events during the wet season in 2022, the water level rose rapidly compared to 2021, indicating that groundwater in the study area was significantly impacted by seasonal effects.

### Water sampling and hydrogeochemical analysis

Groundwater samples of the study were pumped out from 11 groundwater supply facilities that have built-in pump and screened under 72 m from the surface with water tap installed, located in 90–100-m thick fractured hard rock aquifer. A total of 45 samples including the surface water samples were obtained through four times of sampling campaigns in June 2021 (dry season), September 2021 (wet season), January 2022 (dry season), and September 2022 (wet season).

Physicochemical parameters of temperature, EC, DO, TDS, pH, ORP, and salinity were measured by a YSI ProDSS digital instrument (Xlem, USA) after purging groundwater for about 10 min to remove the stagnant water. For the hydrogeochemical analysis, the samples were filtered with a 0.45-μm membrane filter paper and collected in polypropylene bottles. Concentrated nitric acid was used to acidify the samples, resulting in a pH below 2 as a chemical pretreatment for the analysis of cations. Major ions of cation (Na^+^, K^+^, Ca^2+^ and Mg^2+^) and anion (Cl^−^, F^−^, NO_3_^−^, SO_4_^2−^ and HCO_3_^−^) were analyzed using ion chromatography (ICS- 5000; Thermo Scientific Dionex, USA) at the Korea Basic Science Institute (Ochang, South Korea). The quality control of the hydrochemical analysis was confirmed with the charge balance error (CBE = {[Σcations − Σanions]/[Σcations + Σanions]} × 100) of under 10%. Isotopes of oxygen (δ^18^O) and hydrogen (δ^2^H) were measured relative to V-SMOW (Vienna Standard Mean Oceanic Water) with precision of ± 0.2‰ and ± 0.8‰, respectively, using elemental analyzer isotope ratio mass spectrometry (EA-IRMS) at the University of Waterloo, Canada. The composition of sulfate (δ^34^S_SO4_ and δ^18^O_SO4_) isotopes was measured relative to the V-CDT (Vienna Canyon Diablo Troilite) standard at the University of Waterloo with precision of ± 0.3‰ for both isotopes. The radon (^222^Rn) activity of groundwater was measured in the field using RAD7 (radon-in-air monitor, Durridge), which has a measurement accuracy of ± 5%. After connecting the 250-ml bottled sample to RAD7, radon in groundwater was purged out by an internal air pump then passed through the drying tube to remove the moisture of radon gas before circulating in a closed-air loop system. The activity of radon of the sample was determined by counting the alpha-emitting daughters using a charged semiconductor detector.

### Hierarchical clustering analysis (HCA)

Hierarchical clustering analysis (HCA) is one of the most effective clustering mechanisms which is capable of categorizing samples into representative clusters based on their linkage distance, especially in the field of hydrogeochemical research (Güler & Thyne, 2004; Ren et al., 2021; Subba Rao et al., 2020b; Vega et al., 1998). HCA classifies the samples through Euclidean distance to calculate the similarity of the samples, and the Ward linkage rule was used to assess the distance between the clusters, allowing effective classification of the samples (StatSoft Inc., 2004; Cloutier et al., 2008). Additionally, previous studies have compared between various clustering methods and have concluded that HCA was well-suitable for a smaller dataset because of its flexibility on clustering number and lower memory utilization and execution time (Abdalla, 2021; Karthikeyan et al., 2020). In this study, 11 hydrogeochemical input variables which were selected as input data for both dry season and wet season HCA result based on the principal component analysis (PCA) which can help to seek the relative importance of the variables affecting the given dataset and implement the feature selection (Machiwal et al., 2011). Online resource of the PCA results and related description is available in EMS_1 file.

Prior to conducting HCA, the dataset was standardized through subtracting the average value and dividing by the standard deviation of its variables. The standardization process enabled to transform the raw data into a dimensionless dataset in order to eliminate various units and scales throughout the variables (Dillon & Goldstein, 1984; Davis & Sampson, 1986; Tlili-Zrelli et al., 2013). The clusters are formed through connected points with short distances between them and separated by small gaps between clusters. The Euclidean distance method is commonly employed for this purpose presented in Eq. (1) (Wilks, 2011). $D$ stands for the dimension of the data, $d$ is the input variable of the sample, and $k$ is the number of clusters.

1 $$\left|x-y\right|=\sqrt{\sum_{k=1}^D}\left(x_d-y_d\right)^2$$

 

The classification of the samples based on the ward linkage distance displayed in Eq. (2) was visualized through the dendrogram during the dry and wet seasons, respectively (Costello & Osborne, 2005; Irawan et al., 2009). The Ward method begins by initially treating each of the *n* members as its own separate group. Then, in each subsequent step, pairs of groups are merged together. The selection of which pair to merge is based on minimizing the squared error of distances between points (Ward Jr. 1963). ESS is error sum of squares, $K$ is the number of clusters, and $j$ stands for the number of variables. 

2 $$ESS=\sum_{k=1}^K\sum_{x_i\in C_k}\sum_{j=1}^n\left(x_{ij}-{\overline x}_{kj}\right)^2$$

The number of the clusters was determined to three according to the phenon line with distance of 8 in both dry season and wet season results. The procedure of the HCA calculation was performed by using Python 3.7 codes.

### MixSIAR

The proportional contribution of potential sulfur sources in the groundwater samples was estimated through the Bayesian isotope mixing model (MixSIAR) using R statistical package. The MixSIAR model allows the incorporation of fixed and random effects as covariates to account for the variability observed in mixture proportions which gives a differentiation from other previous software of mixing models.

The model generates a logistic prior to distribution based on the Dirichlet distribution. By applying MixSIAR using δ^34^S_SO4_, δ^18^O_SO4_ values, the proportional contribution of the sulfur sources in groundwater can be quantified based on the isotopic compositions of selected end-members including average value, standard deviation, and number of the samples. Since there is no practical influence of the isotope fractionation factor to the model calculation, the values were set to 0. Specific mechanisms and equations are described in Stock et al. (2018).

The proportional contribution of sources (${p}_{k}$) is obtained through the equation of isotope value $j$ of the mixture *i* (${X}_{ij}$) demonstrated in Eq. 3. In this equation, three components are needed to be calculated using the source terms including mean, standard deviation, and number of the samples. The source value (${S}_{jk}$) of isotope *j* is normally distributed with mean (${\mu }_{jk}$) and standard deviation (${\omega }_{jk}^{2}$) values (Eq. 4). The isotope fractionation factor of isotope *j* on source (*C*_*jk*_) is also normally distributed with its mean (${\lambda }_{jk}$) and standard deviation (${\tau }_{jk}^{2}$), but it is filled in with zeros because the microbial activity is not considered (Eq. 5). The third component in Eq. 6 is the residual error (*e*_*ij*_) which has the distribution with mean value of 0 and standard deviation of ${\sigma }_{j}^{2}$. 

3 $$X_{ij}=\sum_{k=1}^kp_k(S_{jk}+C_{jk})+e_{ij}$$

4 $$S_{jk}\sim N\left(\mu_{jk}+\omega_{jk}^2\right)$$

5 $$C_{jk}\sim N\left(\lambda_{jk}+\tau_{jk}^2\right)$$

6 $$e_{ij}\sim N\left(0+\sigma_j^2\right)$$

## Results and discussions

### Hydrogeochemical characteristics of groundwater

#### General hydrogeochemistry

**Fig. 2:**
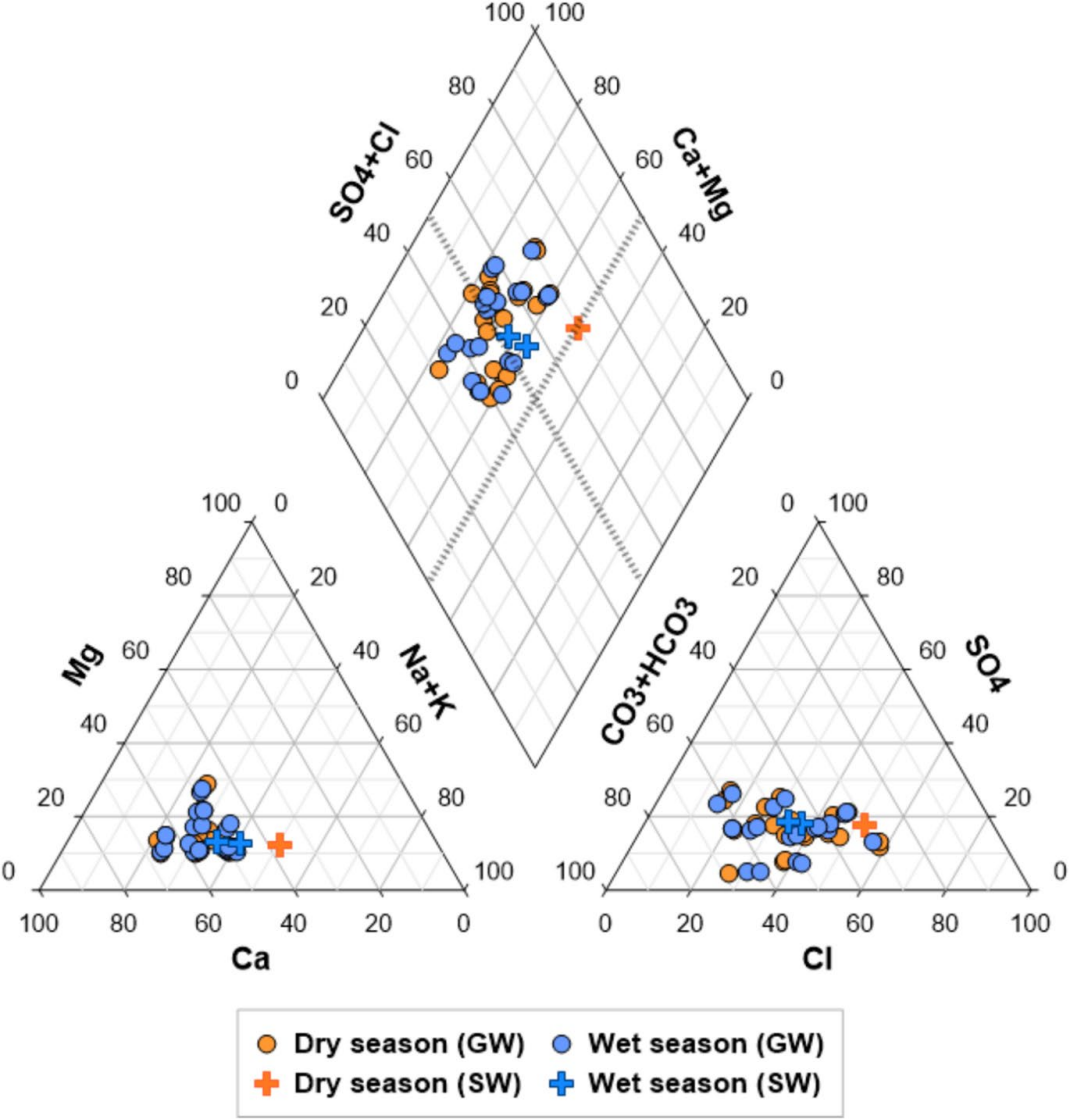
Piper diagram using groundwater and surface water samples collected in both dry and wet seasons

Chemical components of the sampling points are shown by seasons. The Piper diagram reveals the hydrogeochemical evolution of water samples and distinguishes water types based on the proportions of major cations and anions (Fig. 2). The water types of the samples mainly belonged to Ca–Mg–Cl (53.3%) and Ca–HCO_3_ (44.4%), while the surface water in dry season was categorized as Na–Cl type.

The cation and anion ternary diagrams indicated that the chloride proportion is the primary factor influencing the differences in the hydrogeochemical evolution between dry and wet season groundwater. This can be attributed to varying levels of evaporation or recharge processes during these seasons (Eriksson & Khunakasem, 1969; Aishlin & McNamara, 2011).

Pearson’s correlation analysis was performed separately for the dry and wet seasons, as shown in Tables 1 and 2. Pearson’s correlation coefficient (*r*) determines the association between two variables and how effectively one variable can predict the other (Bodrud-Doza et al., 2019; Islam et al., 2018). Change in hydrogeochemical behaviors of natural and anthropogenic factors was observed by the seasonal effect through the analysis. Radon showed a negative correlation with DO in both dry (*r* = − 0.72) and wet (*r* = − 0.63) seasons. EC in the dry season had a positive correlation with Ca^2+^, Mg^2+^, and HCO_3_^−^ and gained higher values with Ca^2+^, Mg^2+^, Na^+^, HCO_3_^−^, Cl^+^, and SO_4_^2−^ in the wet season which are the indicators of a water–rock interaction (Cho & Choo, 2019; Guo et al., 2018; Zhang et al., 2012). Na^+^, HCO_3_^−^, and SO_4_^2−^ also displayed similar patterns during the wet season. F^−^ showed fluctuating correlations particularly with Na^+^ and K^+^ as the season changed. Correlation between NO_3_^−^ and Cl^−^ of anthropogenic indicators presented significance in the dry season (*r* = 0.77) and decreased in the wet season (*r* = 0.52) maintaining its value above 0.50 (Adimalla & Venkatayogi, 2018; Subba Rao et al., 2020b). Further chemical analysis is necessary to investigate specific processes, particularly those involving radon and fluoride.

**Table 1:** Pearson’s correlation coefficients of hydrogeochemical variables in the dry season

	Rn	DO	EC	Ca	K	Mg	Na	HCO3	F	Cl	NO3	SO4
Rn	1											
DO	− 0.72**	1										
EC	0.13	− 0.21	1									
Ca	− 0.02	− 0.16	0.55	1								
K	− 0.27	0.46	0.09	0.02	1							
Mg	0.09	− 0.08	0.62*	0.67*	0.08	1						
Na	− 0.15	0.39	0.36	0.52	0.64*	0.45	1					
HCO3	0.21	− 0.34	0.68*	0.68*	0.02	0.81**	0.36	1				
F	0.37	− 0.43	− 0.35	− 0.18	− 0.30	− 0.22	− 0.53	0.01	1			
Cl	− 0.25	0.28	0.41	0.83**	0.35	0.55	0.73**	0.32	− 0.47	1		
NO3	− 0.24	0.49	0.02	0.44	0.19	0.19	0.56	− 0.16	− 0.41	0.77**	1	
SO4	0.31	− 0.26	0.52	0.56	0.27	0.63*	0.66*	0.68*	− 0.38	0.41	0.05	1

Correlations are significant at *p < 0.05 and **p < 0.01

**Table 2:** Pearson’s correlation coefficients of hydrogeochemical variables in the wet season

	Rn	DO	EC	Ca	K	Mg	Na	HCO3	F	Cl	NO3	SO4
Rn	1											
DO	− 0.63*	1										
EC	0.07	− 0.06	1									
Ca	0.08	− 0.15	0.94*	1								
K	− 0.42	− 0.03	0.25	0.15	1							
Mg	0.17	− 0.07	0.85**	0.74**	0.22	1						
Na	− 0.09	0.22	0.76**	0.67*	0.29	0.57	1					
HCO3	0.09	− 0.15	0.74**	0.76**	0.37	0.78**	0.56	1				
F	0.44	− 0.21	− 0.32	− 0.14	− 0.52	− 0.26	− 0.48	− 0.11	1			
Cl	− 0.10	0.11	0.83**	0.79**	0.09	0.61*	0.62*	0.33	− 0.26	1		
NO3	− 0.09	0.40	0.20	0.08	− 0.26	0.10	0.38	− 0.31	− 0.14	0.52	1	
SO4	0.29	− 0.30	0.75**	0.66*	0.26	0.66*	0.72**	0.64*	− 0.47	0.40	− 0.07	1

Correlations are significant at *p < 0.05 and **p < 0.01

#### Stable isotope compositions of water

The oxygen and hydrogen isotopes of the samples were plotted with the global meteoric water line (Craig, 1961) and local meteoric water line of the Geum watershed area (Jung et al., 2019). Given that the study area is located in East Asia, where precipitation patterns are primarily influenced by the Asian monsoon, it was inferred that the groundwater in this location was significantly influenced by rainfall events during summer monsoon season (Jung et al., 2019; Lee et al., 2003).

**Fig. 3:**
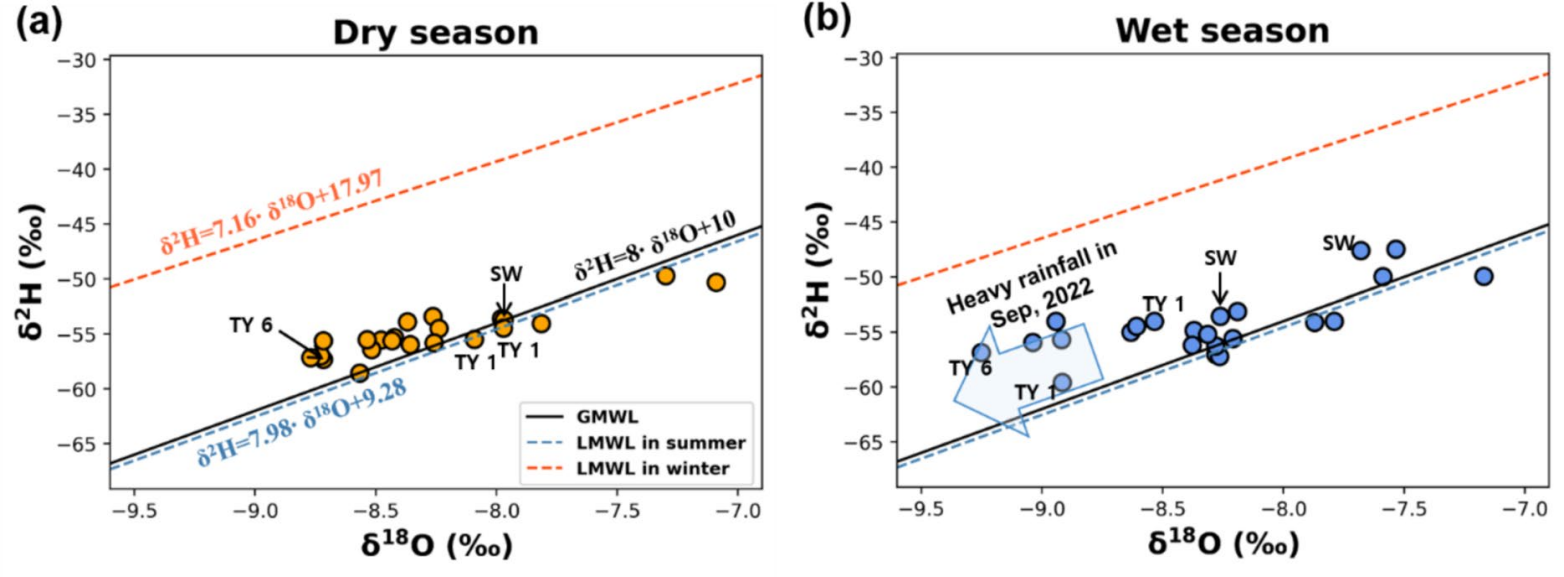
Isotopes of oxygen and hydrogen in water samples compared with global meteoric water line and local meteoric water line of Geum river basin in summer and winter. **a** dry season samples; **b** wet season samples

In the dry season, the oxygen isotope values ranged from − 8.77 to − 7.09‰, and hydrogen isotope values ranged from − 58.55 to − 49.71‰ (Fig. 3a). After the summer rainfall event, the oxygen isotope range shifted from − 9.25 to − 7.17‰, and hydrogen isotope range shifted from − 59.57 to − 47.45‰ (Fig. 3b). The wet season samples from TY- 1, TY- 6, TY- 7, TY- 9, and TY- 11 in November 2022 were notably depleted in heavy oxygen isotopes compared to other samples, which is attributed to recharged heavy rainfall throughout August 2022 (Dansgaard, 1964; Yeh et al., 2014). On the other hand, oxygen isotope values of the SW and TY- 2 samples increased in the wet season, indicating recharge under evaporation processes that originate from the mixing of surface water. Additionally, the enrichment of oxygen isotopes can be caused by the influence of water–rock interactions through the mixing process within deep bedrock groundwater during the wet season (Kendall & McDonnell, 2012; Zhao et al., 2018; Aydin et al., 2020; Ren et al., 2024). The samples that are distributed at the intermediate zone may have been affected by multiple processes of precipitation, surface water, evaporation, and water–rock interaction during the seasonal transition.

### Exploratory data analysis using multivariate statistics

**Fig. 4:**
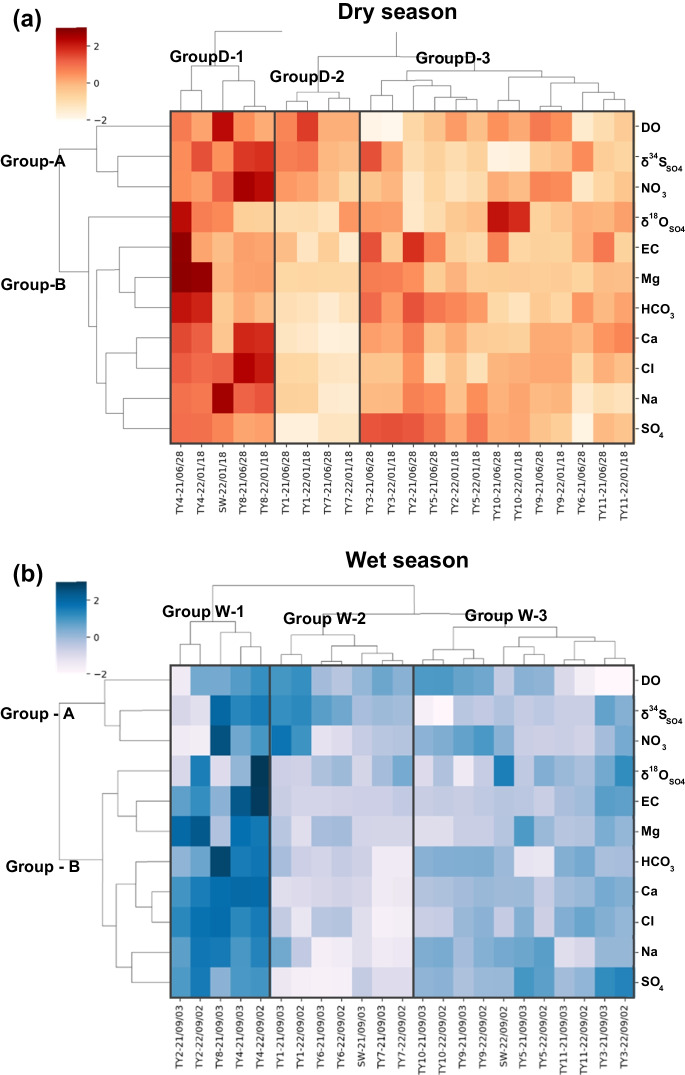
Heat maps presenting the results of Q-mode and R-mode groups through HCA in each season. **a** dry season samples; **b** wet season samples

HCA consists of the Q-mode on the *x*-axis, where the samples are classified into groups, and the R-mode analysis on the *y*-axis, which displays the dendrogram of input variables through a heatmap (Fig. 4a, b). Groundwater and surface water samples were clustered by dry and wet seasons, respectively, to observe the seasonal variation of groundwater within the study area. Average concentration values of the HCA input variables are categorized according to the clustering results of dry and wet seasons in Table 3.

**Table 3:** Average values of input variables by dry and wet season HCA groups

Input variables	Unit	Dry season (*n* = 22)			Wet season (*n* = 23)		
		Group D- 1	Group D- 2	Group D- 3	Group W- 1	Group W- 2	Group W- 3
DO	mg/L	9.13	8.79	6.84	7.74	7.85	6.69
EC	μS/cm	638.28	304.75	500.38	523.66	225.73	339.64
δ^34^S	‰	6.59	5.39	4.13	5.44	5.37	3.95
δ^18^O_SO4_	‰	9.11	4.82	7.58	7.79	5.24	6.05
Ca^2+^	mg/L	48.86	18.58	35.58	55.88	25.64	36.99
Mg^2+^	mg/L	11.58	2.68	5.51	13.35	3.65	5.77
Na^+^	mg/L	33.22	15.30	22.02	31.03	16.68	23.72
HCO_3_^−^	mg/L	101.54	49.78	85.99	141.74	69.62	81.25
Cl^−^	mg/L	78.60	16.33	34.63	61.16	23.44	36.96
NO_3_^−^	mg/L	30.86	17.25	14.15	17.89	15.27	16.31
SO_4_^2−^	mg/L	31.14	8.44	26.95	35.20	10.12	29.50

Variables with minor significance were eliminated using principal component analysis (PCA) (Closs & Nichol, 1975). As a result, the groundwater samples were clustered into 3 groups, and 11 input variables (DO, EC, δ^34^S_SO4_, δ^18^O_SO4_, Ca^2+^, Mg^2+^, Na^+^, HCO_3_^−^, Cl^−^, NO_3_^−^, SO_4_^2−^) were classified into 2 groups in both seasons. The groups formed through R-mode dendrogram were Group-A (DO, δ^34^S_SO4_, NO_3_^−^) and Group-B (δ^18^O_SO4_, EC, Mg^2+^, HCO_3_^−^, Ca^2+^, Cl^−^, Na^+^, SO_4_^2−^).

Z-scores in Group-A appeared to fluctuate within the groups and across seasons, showing a sensitive response to its respective condition by sampling points. Group-B variables consist of most hydrogeochemical indicators, including natural and anthropogenic factors affecting the groundwater (Liu et al., 2023b; Xiao et al., 2022).

In Table 4, the average of fluoride ion and radon concentration which were not included as input variables of the HCA is organized. In contrast to the input variables, the average concentrations of the result of fluoride concentration was as follows: Group D- 3 > Group D- 2 > Group D- 1 and Group W- 2 > Group W- 3 > Group W- 1. Radon concentration result showed Group D- 3 > Group D- 2 > Group D- 1 and Group W- 3 > Group W- 2 > Group W- 1.

**Table 4:** Average values of fluoride and radon concentrations by dry and wet season HCA groups

Variables	Unit	Dry season (*n* = 22)			Wet season (*n* = 23)		
		Group D- 1	Group D- 2	Group D- 3	Group W- 1	Group W- 2	Group W- 3
F^−^	mg/L	0.27	0.45	0.50	0.21	0.63	0.42
^222^Rn	Bq/L	46.80	68.10	213.19	62.57	66.34	97.00

A previous study by Kim and Lee (2023) identified potential mechanisms regulating the chemical composition and quality of groundwater under hydroclimatological scenarios. Dry and rainy season samples from groundwater supply facilities in the Yuseong-gu area were analyzed using method of SOM (self-organizing map). Considering the results presented in this paper, a comprehensive interpretation of the HCA groups was achieved. Groups D- 1 and W- 1 demonstrated groundwater influenced by the water–rock interaction in low-flow and anthropogenic activities (Huang et al., 2023; Hwang & Moon, 2021). Group D- 2 and W- 2 samples appeared to be higher flow groundwater which may be influenced by shallow groundwater and surface water interaction (Banerjee & Ganguly, 2023; Warix et al., 2023). Groups D- 3 and W- 3 were found to be groundwater where higher radon concentrations are observed within the granite bedrock aquifer accompanied by dissolution of fluoride and bicarbonate that originated mainly from the minerals placed at surface of the fractures (Cho & Choo, 2019). Most of the groundwater samples belonged to the group indicating the general groundwater characteristic of the granite bedrock aquifer.

### Effects of natural and anthropogenic processes

#### Natural processes in granite aquifer

To investigate the effects of natural processes, especially water–rock interaction in granite areas, bicarbonate, calcium, and radon were plotted against fluoride concentrations, which are commonly found within granite bedrock (Khan et al., 2024; Kim & Lee, 2023; Srinivasa et al., 2015).

**Fig. 5:**
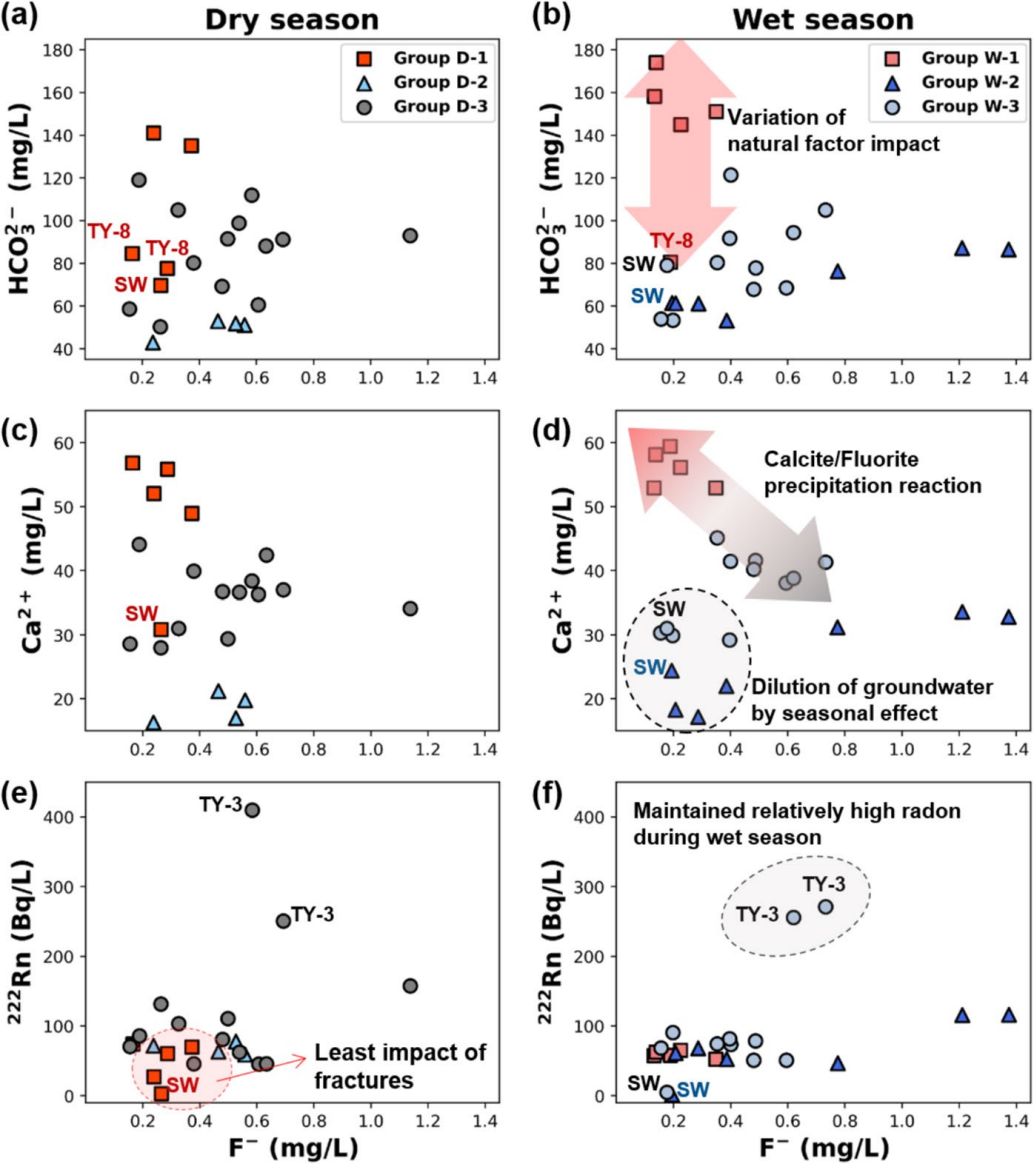
Relationship between bicarbonate, calcium, radon, and fluoride concentrations using labeled samples based on HCA groups. **a, b** Relationship between HCO₃⁻ and F⁻ in the dry and wet seasons, respectively, showing the influence of natural factors; **c, d** Relationship between Ca²⁺ and F⁻ across seasons, highlighting precipitation reactions and seasonal dilution; **e, f** Correlation between ²²²Rn and F⁻ during the dry and wet seasons, indicating fracture effects and sustained radon levels

Compared to the dry season, the TY- 8 sample maintained its separation from the rest of Group W- 1 samples by having a lower bicarbonate concentration of 80.53 mg/L, while other samples had concentrations ranging from 145.74 to 174.32 mg/L (Fig. 5a, b). This suggests that TY- 8 may show distinct hydrogeochemical characteristics within Group W- 1. Also, the elevated bicarbonate concentrations of Group W- 1 samples are attributed to groundwater discharge influenced by water–rock interaction during the wet season. The bicarbonate concentrations showed the impact of water–rock interaction due to the long residence time of groundwater flow. Relatively low concentrations of fluoride may indicate that the dissolution of biotite which is mostly distributed on fracture surface are minor in Group D- 1 and W- 1. This may contribute to the reduced permeability of the groundwater aquifer (Cho & Choo, 2019; Oh et al., 2025).

Group D- 2 and Group W- 2 samples had the lowest concentration range of bicarbonate in both seasons, with 43.02 to 53.03 mg/L and 53.25 to 87.24 mg/L, respectively, indicating that the groundwater was less affected by water–rock interaction. In the wet season, groundwater samples with low bicarbonate concentrations and group transitions in TY- 6 were observed close to the surface water sample. It may imply that Group W- 2 groundwater is possibly impacted by seasonal effect among the groups owing to its short residence time and relatively dynamic groundwater flow (Banks et al., 2009; Kim & Lee, 2023).

Group D- 3 samples encompassed both Group D- 1 and D- 2 ranges. Group W- 3 of wet season became differentiated with Group W- 1 through transition of the groups in wet season samples. The fracture properties at the individual sampling locations such as connectivity or density may have formed this differentiation (Rao et al., 2022). The recharge process from rainwater or surface water infiltration can be controlled by the velocity of the groundwater flow in the fractures causing the variation within Group W- 3 (Cai & Ofterdinger, 2016; He et al., 2023; Meles et al., 2024).

Calcite precipitation and fluoride release reactions in groundwater interacting with granite bedrock were examined through the relationship between calcium and fluoride ions. In the dry season, the groups generally seemed to be separated by calcium concentration, with Group D- 1 > Group D- 3 > Group D- 2, and samples with relatively high fluoride concentrations were mainly observed in Group D- 3 (Fig. 5c). Despite the rainfall events, there were no notable changes in the calcium concentration range within each group. In contrast, fluoride concentrations exhibited an evolving pattern across the groups, by Group W- 2 > Group W- 3 > Group W- 1, presenting a contrasting trend of calcium (Fig. 5d).

According to the batch experiment of Chae et al. (2006), the paper has concluded that the interaction with Ca-bearing plagioclase contributes the sufficient amount of calcium ions to groundwater but restricts the increase of calcium and fluoride concentration through chemical reaction of fluorite (CaF_2_) precipitation in the granite bedrock groundwater system. The formation of calcite is the main factor behind the accumulation of fluoride ions in groundwater, and there is a potential for additional rise in fluoride concentrations because of the dissolution of fluorite (Al Sabti et al., 2023; Xu et al., 2023).

Both Group D- 1 and Group W- 1 well exhibited the phenomenon suggested in the experiment by having the highest calcium concentrations and the lowest fluoride concentrations among all the groups in their respective season due to the limited source of fluoride ions. Group W- 2 samples did not show the fluorite precipitation reaction indicating the different origin of fluoride with Group W- 3 such as shallow weathered layer or soil water (Subba Rao et al., 2020a). Compared to Group D- 3 in the dry season, samples with calcium and fluoride concentrations higher than those of surface water exhibited a declining trend along with the Group W- 1 samples. Samples which had comparable concentrations to the surface water were likely located at the fracture zones dominated by the dilution effect during the wet season (Banks et al., 2009; Wang et al., 2015; Warix et al., 2023).

Investigation of radon and fluoride examined the influence of fractures release and mobilize into the groundwater system especially in granite aquifers (Cho & Choo, 2019; Oh et al., 2025). The results for group D- 1 and group W- 1 showed that the groundwater belonging to these groups was not influenced by granitic fractures through possessing the lowest range of radon concentration in both seasons (Fig. 5e, f). Group D- 2 samples generally showed higher radon concentrations than group D- 1, but decrease in group W- 2 was observed compared to dry season except TY- 6 which belonged to group D- 3. This fluctuating radon concentration and group change in group D- 2 and group W- 2 has demonstrated higher flow groundwater in relatively permeable fractures. However, surface water interaction did not solely form chemical concentrations of bedrock groundwater in the Yuseong-gu area (Kim & Lee, 2023). Radon alone did not clearly show the direct interaction with surface water, as the samples were collected from a deep hard rock aquifer located far from surface water sources. (Gu et al., 2024; Johnson et al., 2024). Group D- 3 samples generally had higher radon concentrations than other groups (45.25 ~ 409.88 Bq/L). Group W- 3 samples appeared to have an overall decrease in radon concentrations, but also displayed samples with higher concentration than other groups (4.68 ~ 271.03 Bq/L). Throughout the seasons, dilution of radon concentrations mainly occurred in the wet season. However, TY- 3 samples from group D- 3 and group W- 3 consistently maintained their abnormally high radon concentrations, ranging between 250.59 ~ 409.88 Bq/L, along with higher fluoride concentrations of 0.58 ~ 0.73 mg/L, compared to other samples. These evidences suggested that groups D- 3 and W- 3 possessed samples closely in contact with uranium ore body and biotite minerals in fractured granite aquifer and samples significantly affected by seasonal effect (Lachassagne et al., 2021; Bhavya et al., 2023; Surbramaniyan and Elango 2024).

#### Effects of anthropogenic activities

**Fig. 6:**
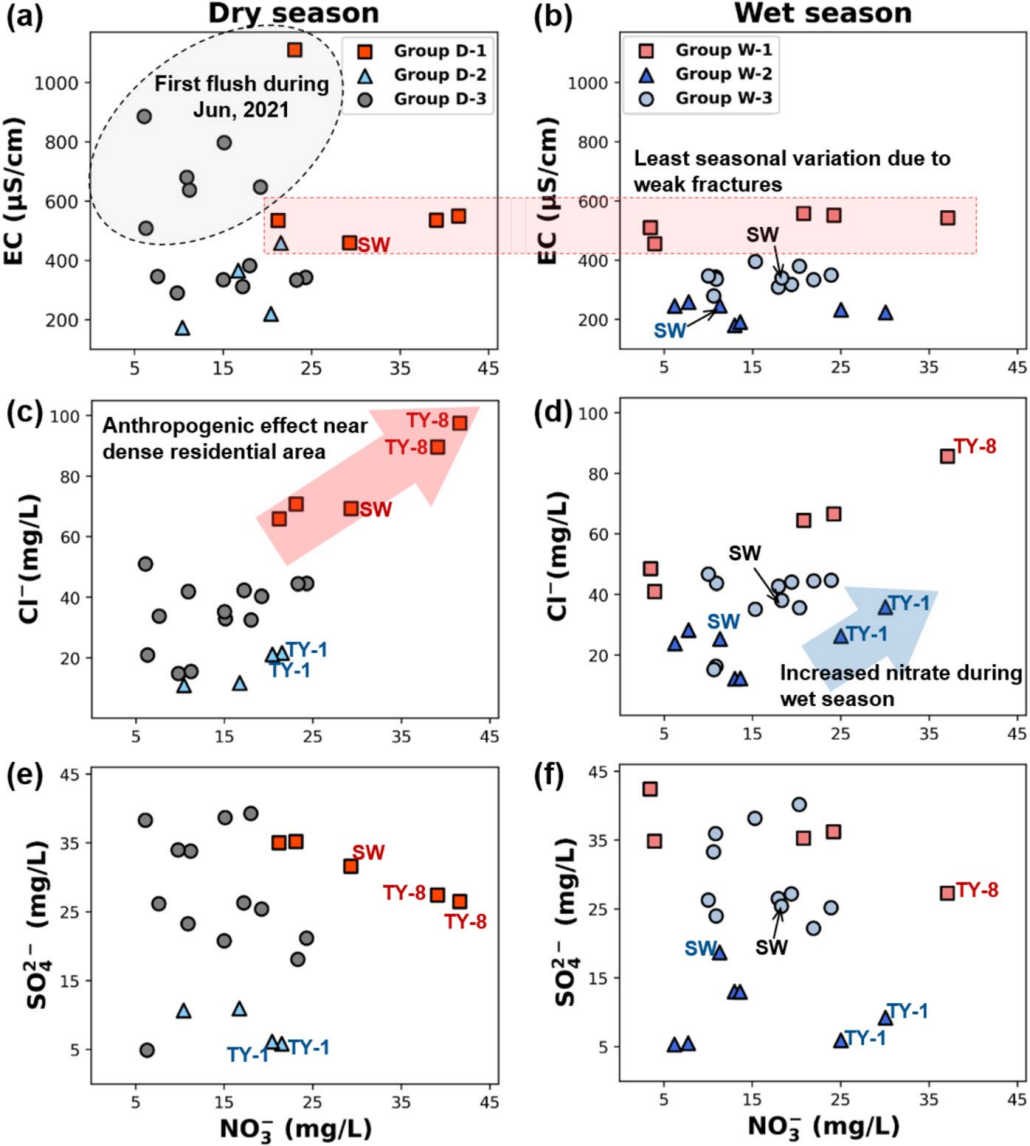
Relationship between EC, chloride, sulfate, and nitrate concentrations using labeled samples based on HCA groups. **a, b** Relationship between EC and NO₃⁻ in the dry and wet seasons, respectively, showing the effect of initial flushing and fracture-controlled transport; **c, d** Relationship between Cl⁻ and NO₃⁻ across seasons, indicating anthropogenic influence and seasonal nitrate increase; **e, f** Relationship between SO₄²⁻ and NO₃⁻ during the dry and wet seasons, reflecting co-contamination trends from surface activities

To observe the influence of anthropogenic contamination separately from natural effects, nitrate was used along with EC, chloride, and sulfate concentrations. The EC concentrations of the dry season samples were not classified by the HCA groups but showed differences between the two sampling campaigns (Fig. 6a). Rainfall during the dry season has begun in middle of May 2021. Precipitation during the dry season might have increased the EC concentration in the June 2021 groundwater. As the recharge process initiated by rainwater continues, the release of ions into the groundwater can occur depending on the presence of dissolved CO_2_ and carbonic acid (Elango & Kannan, 2007; Obiri-Nyarko et al., 2023). Furthermore, “first flush” phenomenon can also contribute to the increasing EC concentration during the dry season as a human-induced contamination factor (Gao et al., 2023; Niazkar et al., 2024). Compared to the dry season, wet season samples showed dilution effect particularly in group W- 3 through decreasing trend of EC concentrations (Fig. 6b). In contrast, minimal seasonal variation between groups D- 1 and W- 1 was observed due to the disconnection from the shallower groundwater in less developed fractures.

Although Yuseong-gu groundwater facilities were reported as an area less influenced by anthropogenic activities than other districts in Daejeon, the possibility of contamination from domestic water has been suggested (Jeong, 2001; Jeong, 2003). Group D- 1 was observed to contain samples possessing both highest EC (1111 μS/cm) and nitrate concentration (41.65 mg/L) within the study area indicating relatively contaminated with anthropogenic sources. Compared to other groups, the variation in EC concentrations between groups D- 1 and W- 1 did not show a significant seasonal effect.

**Fig. 7:**
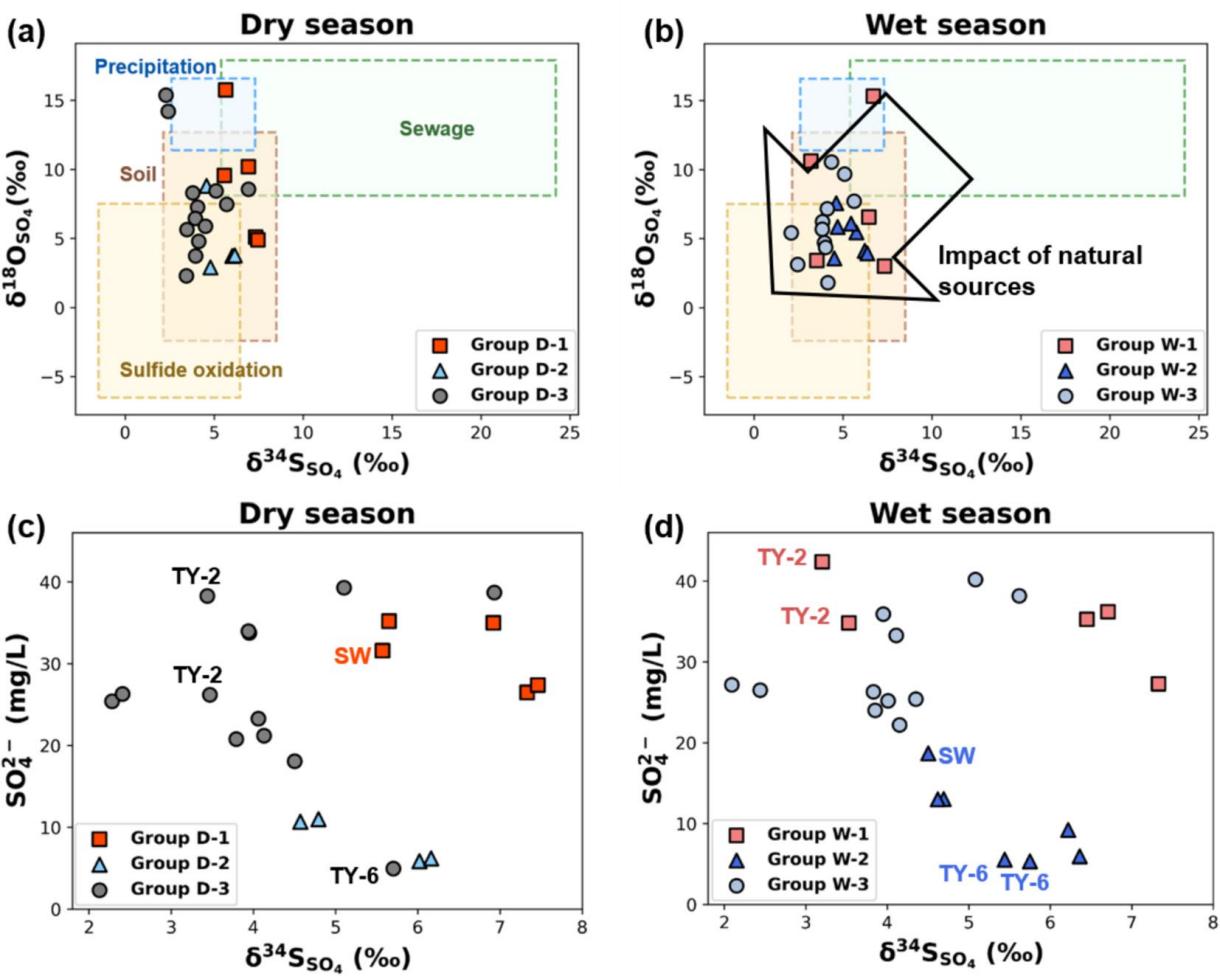
δ^34^S_SO4_ and δ^18^O_SO4_ ranges of potential sulfur sources including natural and anthropogenic factors in the Yuseong-gu groundwater system, with sulfate concentration of the water samples. **a, b** δ³⁴S–SO₄²⁻ and δ¹⁸O–SO₄²⁻ relationships during the dry and wet seasons, respectively, indicating contributions from various natural and anthropogenic sources; **c, d** Relationship between sulfate concentration and δ³⁴S–SO₄²⁻ in the dry and wet seasons, respectively

The chloride result in dry season presented positive correlation with nitrate concentration and well displayed group D- 1 as relatively contaminated groundwater (Fig. 6c). By interpreting the nitrate results in combination with bicarbonate results depicted in Fig. 7a, the TY- 8 sampling point is identified as groundwater situated in an area where contamination has occurred, surrounded by a densely populated apartment complex producing domestic wastewater (Jin et al., 2004). TY- 1 in group W- 2 also showed the impact of nitrate and chloride contamination due to the permeable aquifer which allowed the infiltration or discharge of the polluted water through the seasonal effect (Alsabti et al., 2023; Pitt et al., 2023) (Fig. 6d). The rest of the groundwater samples in group W- 2 were dominated by dilution rather than anthropogenic contamination.

Sulfate is known to be found in both natural and anthropogenic sources, leading a poor relationship with nitrate ions. However, the possibility of sulfate from anthropogenic contamination is suggested by groups D- 1 and W- 1 (Fig. 6e, f). Natural processes such as mineral oxidation and the input of soil-derived water may hinder sewage signatures and obscure direct relationships between individual ions (Bao et al., 2022; Gugulothu et al., 2022; Bakche et al., 2024). In addition, nitrate and sulfate behave differently in the subsurface environment. According to the unclear distinction of the groups during the wet season, further source identification can be interpreted using the stable isotopes of sulfur and oxygen to investigate seasonal variation of both natural and anthropogenic processes in the groundwater system.

### Potential sulfur sources in the study area

Four potential sulfur sources in the Yuseong-gu area were identified using δ^34^S_SO4_ and δ^18^O_SO4_ to investigate the seasonal variation of groundwater by tracing sulfate originating from natural and anthropogenic processes (Fig. 7a, b). The potential sulfur sources of groundwater and surface water were found to be (1) precipitation, (2) sewage, (3) soil, and (4) sulfide oxidation. Statistical information of these sulfur source ranges is presented in Table 5.

**Table 5:** Selected sulfur origins in groundwater as determined from published information

Sources	δ^34^S_SO4_		δ^18^O_SO4_		Reference
	Mean	SD	Mean	SD	
Precipitation	4.87	1.38	14.57	1.55	Yu and Park (2004); Lim et al. (2012)
Sewage	9.93	4.05	10.68	2.29	Bottrell et al. (2008); Otero et al. (2008); Shin et al. (2015)
Soil	5.21	1.72	7.06	4.41	Mayer et al. (1995); Zhang et al. (2015)
Sulfide oxidation	1.96	2.23	0.82	5.49	Park et al. (1991); Jezierski et al. (2006); Lipfert et al. (2007)

Biogenic effects of sulfur-reducing bacteria (20 ~ 56‰), which are known to use sulfates as an electron acceptor in respiration, especially in anaerobic conditions, were within a range that could not affect the groundwater at the study area. However, since the groundwater system at the study area was determined to be aerobic by DO (3.76 ~ 11.89 mg/L), biogenic factors were not considered sulfur sources in this research. Evaporite was also excluded as candidate source because the evaporite weathering process is reported to be not considered as a significant factor in the Daejeon granite area (Cho & Choo, 2019; Jung et al., 2019; Lee et al., 2019).

Sulfur from precipitation was collected to investigate the input of atmospheric pollutants into the groundwater and its relationship with seasonal effects. The δ^34^S_SO4_ range varied between 2.6 and 7.3‰ (*n* = 27), and δ^18^O_SO4_ values were distributed within 11.4 ~ 16.6‰ (*n* = 18) (Lim et al., 2012; Yu & Park, 2004). The obtained sewage source samples had δ^34^S_SO4_ ranges of 5.4 ~ 24.2‰ (*n* = 23) and δ^18^O_SO4_ values ranging from 8.1 to 17.9‰ (*n* = 21). These samples were gathered from sulfate-contaminated water in cities, including Daejeon, and from sewage treatment plant water samples (Bottrell et al., 2008; Otero et al., 2008; Shin et al., 2015). Sulfur in soil is bonded with carbon and releases sulfate through organic sulfur oxidation in soil. The ranges of δ^34^S_SO4_ and δ^18^O_SO4_ in soil water are 2.14 ~ 8.5‰ (*n* = 12) and − 2.4 ~ 12.7‰ (*n* = 10), respectively (Mayer et al., 1995; Zhang et al., 2015). Sulfide oxidation in minerals including pyrite samples was mainly collected within the granite bedrock aquifer comparable to the groundwater system in Yuseong-gu. The δ^34^S_SO4_ values were within − 1.5 ~ 6.46‰ (*n* = 30) and δ^18^O_SO4_ values between − 6.53 and 7.52‰ (*n* = 8) (Jezierski et al., 2006; Lipfert et al., 2007; Park et al., 1991).

The δ^34^S_SO4_ values of dry season samples were ranged from 2.28 to 7.46‰, while the wet season samples varied between 2.09 and 7.33‰, showing a relatively lower range, which can be inferred as the input of additional natural sources of soil and sulfide oxidation in the overall groups. The range of δ^18^O_SO4_ of dry season was 2.31 ~ 15.76‰ and wet season samples spanned from 1.81 to 15.34‰. Although these ranges did not display noticeable changes between the dry and wet seasons due to mixed multiple sources, the groups classified by HCA demonstrated shifts within the potential sulfur sources. This result showed agreement with the hydrogeochemical results of natural and anthropogenic impacts during the seasonal changes (Gonfiantini et al., 1998; Kaown et al., 2009). Group D- 1 was mainly distributed within the ranges of precipitation and sewage. TY- 8 samples were observed to be located closer to the sulfur isotope range of sewage than other samples which matched to the result of anthropogenic effects in Fig. 6.

In rainy season, broader sulfur isotope values were observed in group W- 1 as the TY- 2 samples shifted to its group possessing the highest sulfate concentration (42.38 mg/L) (Fig. 7d). This result indicated that multiple sources from both natural and anthropogenic factors can be found in a fractured aquifer. Samples in groups D- 2 and W- 2 were mostly located near sulfide oxidation; soil source ranges with the lowest sulfate concentration ranges in both seasons. Groups D- 3 and W- 3 also were affected by sulfide oxidation and soil sources, but several samples in the wet season evolved towards the sulfide oxidation zone. Furthermore, no notable increasing trend of the between sulfur isotope values and sulfate concentrations of groundwater samples was observed indicating less biogenic effects (Miao et al., 2012; Puig et al., 2013).

The multiple sulfur sources affecting the groundwater caused overlapping ranges between the groups. Further mixing processes were observed during the rainy season, but agreement with previous hydrogeochemical analysis results was also found. Additional research is suggested to further analyze the factors related to natural and artificial factors in greater detail.

Estimation of natural and anthropogenic sulfur source contributions through Bayesian isotope mixing model.

**Fig. 8:**
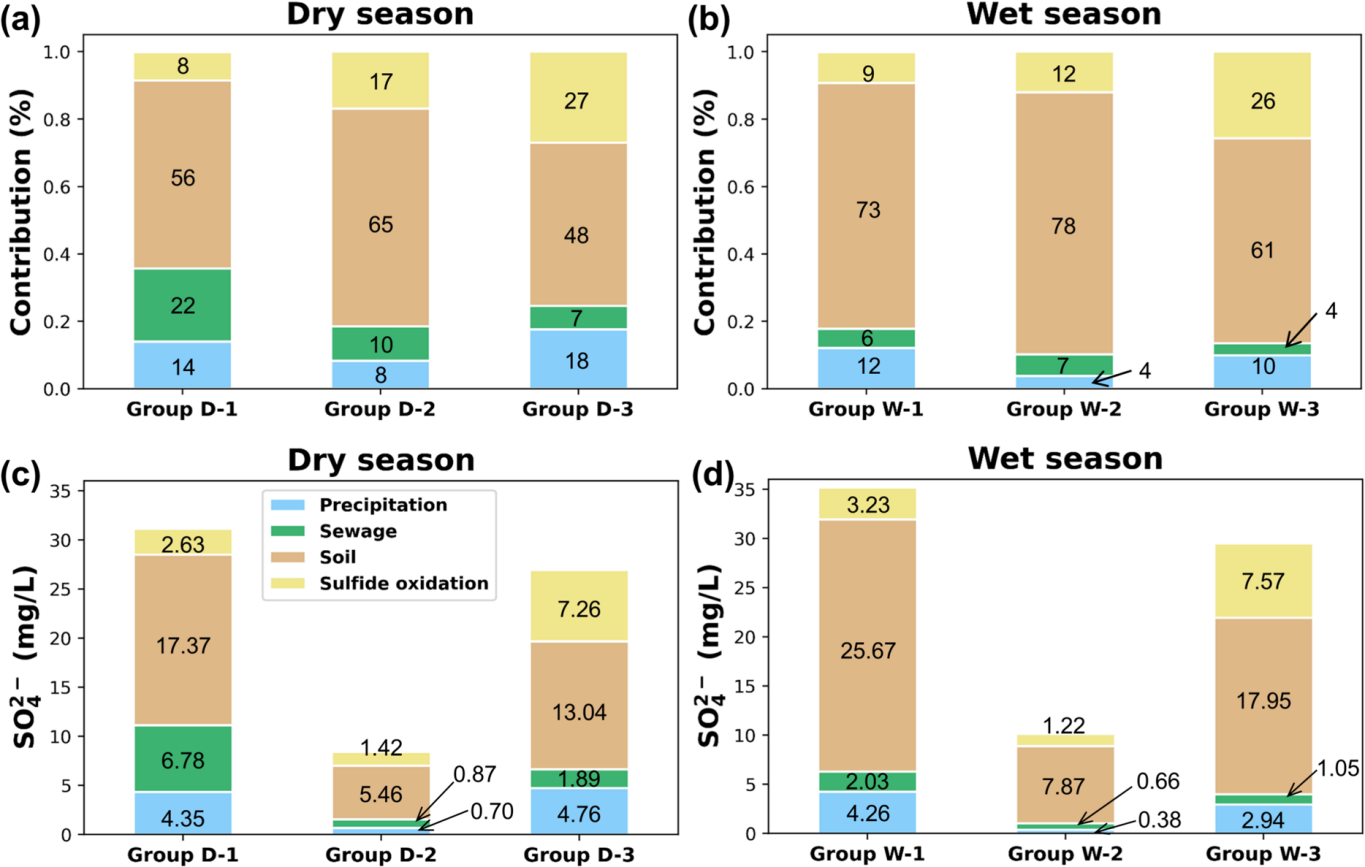
Proportional contributions of sulfur sources and average sulfate concentration of each groups considering proportional contributions in dry and wet seasons. **a, b** Estimated contributions (%) of potential SO₄²⁻ sources (precipitation, sewage, soil, and sulfide oxidation) during the dry and wet seasons, respectively, for each hydrochemical group; **c, d** SO₄²⁻ concentrations and their respective source partitioning in the dry and wet seasons

The estimated proportion contributions of the identified sulfur sources were organized by the average values of the groups in each season (Fig. 8a, b). Sulfates from precipitation indicated atmospheric decomposition which reflect the SO_X_ air pollutants of combusted fossil fuels and seasonal effect of rainfall (Li et al., 2006; Porowski et al., 2019). The overall contribution of precipitation in dry season groups decreased during the wet season. In South Korea, air pollutant emissions were higher during cold seasons compared to warm seasons primarily due to a combination of domestic heating and meteorological conditions as reported throughout the previous studies including long-term monitoring results of metropolitan cities (So et al., 1996; Lim et al., 2012; Ray & Kim, 2014). Atmospheric pollutants can be regarded as one of the anthropogenic factors in the groundwater system during the dry season, but it became a minor factor in the wet season due to reduced emissions.

Sewage showed the highest contribution value in group D- 1 (12.48%) but decreased during the wet season (8.30%). This was consistent with the results of anthropogenic impacts shown in Fig. 6, indicating that group D- 1, which included samples located near dense residential areas (TY- 8), was influenced by human-induced contaminants, particularly in the dry season, as evidenced by higher chloride and nitrite concentrations than other groups. However, the decline in sewage contribution during the wet season indicated that natural factors, including rainfall, played a more significant role than anthropogenic influences.

Among the sulfur sources, soil was the dominant source in all groups indicating that a natural factor was mainly controlling the groundwater system of Yuseong-gu area. The study area was expected to undergo groundwater recharge primarily through infiltration, especially through the unsaturated soil layer during the wet season. It is due to the large area of forest and grassland surrounding the study area, accelerating continuous infiltration or discharge process of groundwater affected by soil, resulting in the arrival to the deeper aquifer (Adane et al., 2018; Chen et al., 2024a). Furthermore, the origins of soil sulfate are mostly formed by the combination of atmospheric decomposition, hydrolyzed organic sulfates, and mineralization of carbon-bonded sulfur, which increases with depth, forming intermediate range (Mayer et al., 1995). There has been a significant seasonal variation in soil contribution between group D- 1 (50.04%) and group W- 1 (74.00%). During the wet season, the increased input from natural sources such as soil and sulfide oxidation may lead to a decrease in δ^34^S_SO4_ and δ^18^O_SO4_ values of group D- 1 samples, which originally had the highest values due to the influence of anthropogenic sources. Group W- 2 appeared to be dominated by the soil source (73.84%) due to interaction with the shallower part of the aquifer, which contains soil water, rather than deeper groundwater during rainfall.

Sulfide oxidation in S-bearing minerals appeared to be a significant natural factor in the fractured granite groundwater of group D- 3 (31.35%) and showed minimal effect in group W- 1 (10.22%). Sulfide oxidation mostly occur below the water table where sulfide mineral deposits are in contact with oxygen-rich groundwater or with intense redox gradients (Hamilton, 1998; Leybourne et al., 2006; Nicholson, 1994). MixSIAR analysis has shown that the fracture groundwater of group D- 3 and group W- 3 represent a favorable condition for oxidative weathering in the study area. Moreover, South Korean studies frequently have reported pyrite oxidation in fractured aquifers (Chae et al., 2001, Banks et al., 2021; Ju et al., 2023). A decrease in the contribution of sulfide oxidation was observed due to infiltration caused by rainfall, which increased the soil source contribution to the study area groundwater rather than the sulfide oxidation source. Upon converting the contribution ratios into average sulfate concentrations for each group, it was observed that overall sulfate concentrations increased during the wet season (Fig. 8d). Particularly, the soil origin of group W- 1 showed the greatest increase, confirming once again that group W- 1 was significantly influenced by the mixture of natural and anthropogenic factors during the recharge or discharge process in the wet season. Conversely, groups D- 2 and W- 2 were identified as groundwater with the least influence from water–rock interaction and artificial contamination.

### Hydrogeochemical conceptual model based on the groups

**Fig. 9:**
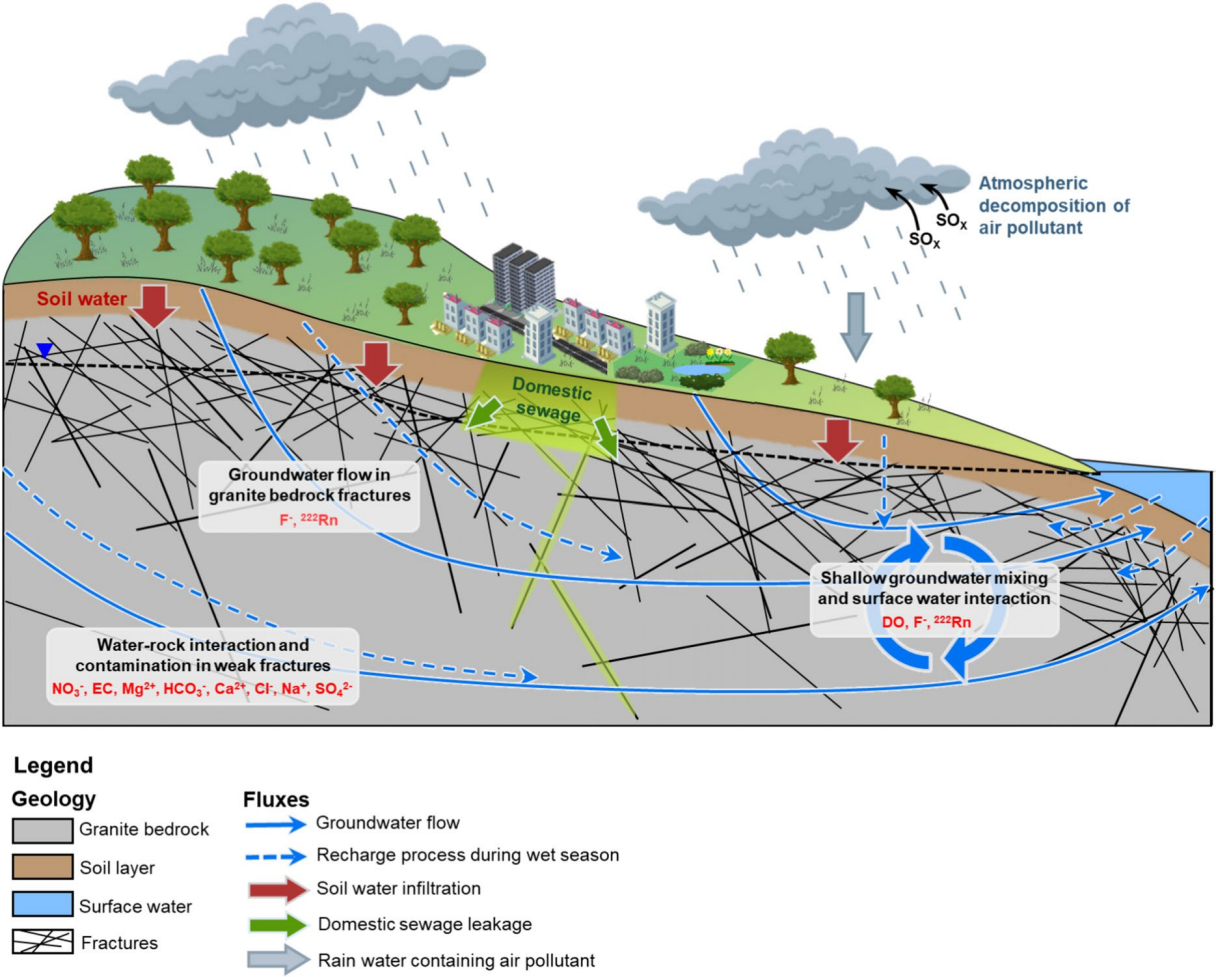
Conceptual model displaying behaviors of natural and anthropogenic factors identified in the Yuseong-gu granite groundwater system using representative hydrogeochemical variables of HCA groups

The identified natural and anthropogenic factors influencing the groundwater system were depicted through a conceptual model based on the HCA groups, hydrogeochemical results, and groundwater movement influenced by climatic impacts (Fig. 9).

Group D- 1 groundwater samples with low velocity are due to the narrower and fewer flow paths. The relatively low residence time of the groundwater induces water–rock interaction, leading to higher concentrations of hydrogeochemical components from minerals compared to other groups. The sulfur isotope mixing model results suggested that group D- 1 was most affected by anthropogenic effects from the surrounding residential area. Contaminants in deep fractured aquifers are often found to be transferred through the fractures from the surface (Khatri et al., 2015; Bondu et al., 2017; El Alfy et al., 2017; Burri et al., 2019). Group W- 1 groundwater samples were determined to be recharged by nearby groundwater. It is also influenced by natural and anthropogenic factors, providing additional chemical concentrations to the groundwater instead of showing dilution during the wet season.

Group D- 2 samples generally showed groundwater influenced less by natural and anthropogenic factors. The fresh groundwater in granite bedrock aquifer is known to be mixed with recharged modern meteoric water which usually originates from the surface zone and mostly remain in shallow aquifer. Group W- 2 was observed to be influenced by fractures and soil water and expected to have a shorter residence time, which would inhibit the water–rock interaction process.

Both group D- 3 and group W- 3 showed the influence of fractures possessing granite rock through radon and fluoride concentrations. It displayed a broader range of the hydrogeochemical characteristics between those of the other HCA groups depending on the fracture properties in the groundwater sampling points. The seasonal effect observed in group W- 3 also varied by locations, suggesting that fracture properties are deeply involved in the recharge or discharge process of groundwater during the wet season.

## Conclusion

This study investigated the impacts within the granite groundwater system by analyzing seasonal variations using environmental tracers to obtain detailed hydrogeochemical insights. Groundwater mixing and hydraulic conditions, caused by fractured crystalline bedrock, were evaluated by identifying both natural and anthropogenic factors. Major sources and hydrogeochemical processes in groundwater were identified by integrating sulfur isotopes with radon and fluoride, enhancing the interpretation of natural influences on the system.

The findings of this research are as follows:

1. HCA classified groundwater into three groups in both dry (D) and wet (W) seasons. Group D- 1 and W- 1 represented groundwater in low flow with long residence time and highly affected by natural and anthropogenic factors. Groups D- 2 and W- 2 were higher flow groundwater influenced by shallow groundwater and surface water interaction. Groups D- 3 and W- 3 showed groundwater with higher radon concentration, flowing through fractures of the granite bedrock aquifer, representing the general groundwater type in the study area.
2. In areas with well-developed granitic fractures, dissolution of geogenic contaminants such as fluoride and radon was observed during the wet season. In residential areas with nearly closed fracture systems, seasonal variations were limited due to restricted groundwater flow. In contrast, in higher flow regions, fresh groundwater resulted from shallow groundwater and surface water mixing process, demonstrating significant seasonal changes.
3. The sulfur isotope enabled the quantitative assessment by calculating contributions of natural and anthropogenic sulfur origins to the groundwater system. Identified major sulfur sources were precipitation (~ 14%), sewage (~ 22%), soil (~ 78%), and sulfide oxidation (~ 27%). Natural processes, such as sulfide oxidation, contributed the most in fractured zones, where sulfide mineral deposits are in contact with oxygen-rich groundwater. Especially in the wet season, soil appeared to be a significant source in the study site indicating the effect of infiltration along with increasing the mixing process between the multiple sources.
4. A hydrogeochemical conceptual model was developed to enhance the understanding of hydrogeochemical processes. The interaction of natural and anthropogenic sources and flow paths was shown with the seasonal effects. This model provides a comprehensive interpretation of hydrogeochemical behaviors within the granite bedrock groundwater system.

Seasonal effects of groundwater varied mostly by fracture properties and accelerated mixing of multiple sources hindering hydrogeochemical identification. As the seasonal patterns become unstable due to climate change, investigating the groundwater system will become more challenging. This study demonstrates how integrating statistical methods with isotopic tracers provides a specialized approach for identifying hydrogeochemical processes in granite-fractured groundwater, an essential step in monitoring groundwater quality and preserving drinking water resources. This study addresses several limitations, including the absence of information on subsurface heterogeneity, mixing process of the multiple sources during the wet season, and uncertainties in the MixSIAR model arising from source data obtained from previous literature. These limitations can be further investigated in future studies by obtaining advanced source data including isotopic data near sampling points of the study site.

## Supplementary Information

Below is the link to the electronic supplementary material. 

ESM1 (DOCX 266 KB)

## Data Availability

No datasets were generated or analysed during the current study.
