# Supplementary material for: Natural and anthropogenic factors controlling hydrogeochemical processes in a fractured granite bedrock aquifer, Korea
Source: Environ Monit Assess. 2025 Apr 30;197(5):613. doi: 10.1007/s10661-025-14037-y (PMC12043750; doi:10.1007/s10661-025-14037-y)
Supplement: Supplementary file 1 — (DOCX 266 KB) [file 10661_2025_14037_MOESM1_ESM.docx]

**Environmental Monitoring and Assessment – Supplementary material**

**Table S1 Statistical summary of hydrogeochemical components collected during four sampling campaigns (Jun-29-2021, Sep-03-2021, Jan-18-2022, Sep-02-2022)**

| **Variables** | **Unit** | **Mean** | **Median** | **Minimum** | **Maximum** | **SD** |
| --- | --- | --- | --- | --- | --- | --- |
| **EC** | μS/cm | 418.88 | 347.00 | 173.70 | 1111.00 | 192.48 |
| **pH** | - | 6.48 | 6.47 | 5.66 | 7.04 | 0.31 |
| **Eh** | mV | 392.46 | 391.70 | 287.50 | 446.20 | 29.99 |
| **DO** | mg/L | 7.49 | 7.86 | 3.76 | 11.89 | 1.76 |
| **Ca^2+^** | mg/L | 36.59 | 36.62 | 16.26 | 59.41 | 11.87 |
| **K^+^** | mg/L | 2.15 | 1.46 | 0.85 | 9.82 | 1.97 |
| **Mg^2+^** | mg/L | 6.58 | 4.58 | 2.63 | 19.74 | 4.48 |
| **Na^+^** | mg/L | 23.25 | 23.59 | 12.05 | 42.00 | 6.90 |
| **HCO_3_^-^** | mg/L | 86.98 | 80.41 | 43.03 | 173.74 | 31.85 |
| **F^-^** | mg/L | 0.44 | 0.38 | 0.13 | 1.37 | 0.28 |
| **Cl^-^** | mg/L | 39.66 | 38.08 | 10.86 | 97.53 | 21.14 |
| **NO_3_^-^** | mg/L | 17.40 | 17.22 | 3.44 | 41.65 | 8.92 |
| **SO_4_^2-^** | mg/L | 24.69 | 26.28 | 4.94 | 42.38 | 11.22 |
| **δ^34^S** | ‰ | 4.81 | 4.57 | 2.09 | 7.46 | 1.42 |
| **^18^O_SO4_** | ‰ | 6.79 | 5.89 | 1.81 | 15.76 | 3.49 |
| **δ^18^O** | ‰ | -8.28 | -8.31 | -9.25 | -7.09 | 0.48 |
| **δ^2^H** | ‰ | -54.58 | -55.18 | -59.57 | -47.45 | 2.58 |
| **^222^Rn** | Bq/L | 88.13 | 68.26 | 1.51 | 409.88 | 75.04 |

Principal component analysis (PCA) is a well-known multivariate statistical data analysis technique for analyzing hydrogeochemical datasets with high dimensions. The success of extracting related variables and inferring the underlying hydrogeochemical characteristics such as natural or anthropogenic processes of groundwater has been reported through previous studies (Helena et al., 2000; Güler et al., 2002; Thyne et al., 2004; Güler et al., 2012).

Through PCA, principal components (PCs) can be obtained to reduce dimensionality of the dataset by new variables that are consisted with linear combinations of the original variables. The calculation of PCs can be done by extracting the eigenvalues and eigenvectors (loadings) of covariance matrix through multiplying the original variables with the eigenvector. The equation of covariance matrix is in Eq. S1, and the process of eigen-decomposition is in Eq. S2. $X$ is the original data matrix, $\bar{x}$ is the mean matrix of $X$, *n* is the number of features representing the dimension of the dataset, *v* is the eigenvector matrix and *L* is the eigenvalue matrix.

(Eq. S1)

$$\Sigma=\frac{1}{n-1}\left( \left( X-\bar{x} \right)^{T}\left( X-\bar{x} \right) \right)$$

(Eq. S2)

$$\Sigma V=LV$$

The variance associated with the principal components is measured by their eigenvalues, loadings indicate the extent to which the original variables contribute to the principal component, and the transformed observations for each individual are referred to as scores (Vega et al. 1998; Helena et al. 2000; Kumar et al., 2009). The principal component matrix can be obtained by Eq. S3 using the eigenvalue matrix ($A^{T}$) multiplied with *X*.

(Eq. S3)

$$Z=A^{T}X$$

In this study, after eliminating variables with minor significance (Closs and Nichol, 1975), DO, EC, δ^34^S_SO4_, δ^18^O_SO4_, Ca^2+^, Mg^2+^, Na^+^, HCO_3_^2-^, Cl^-^, NO_3_^-^, SO_4_^2-^ were selected as input variables for the PCA result. The combined explained variance ratio of PC1 and PC2 accounted for 65.4%, demonstrating the reflected proportion of the original dataset’s variance. Based on the PCA result, clustering technique was applied to investigate the sulfur sources in groundwater of the study area.


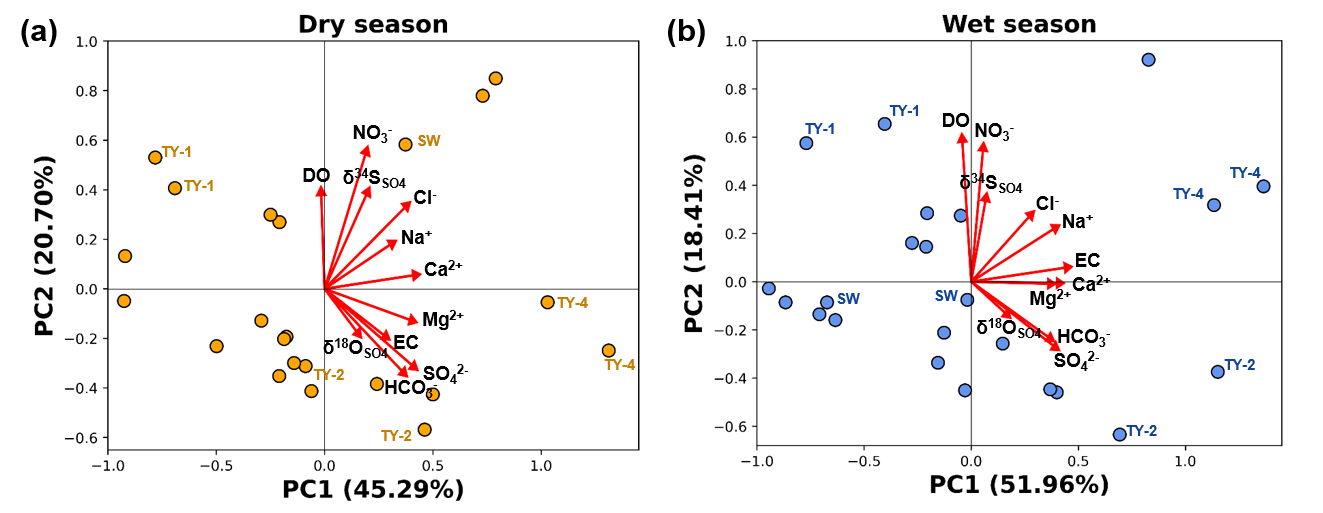


**Fig. S2 Score plot of PC1 and PC2 along with loadings of input variables by dry and wet season.**

**
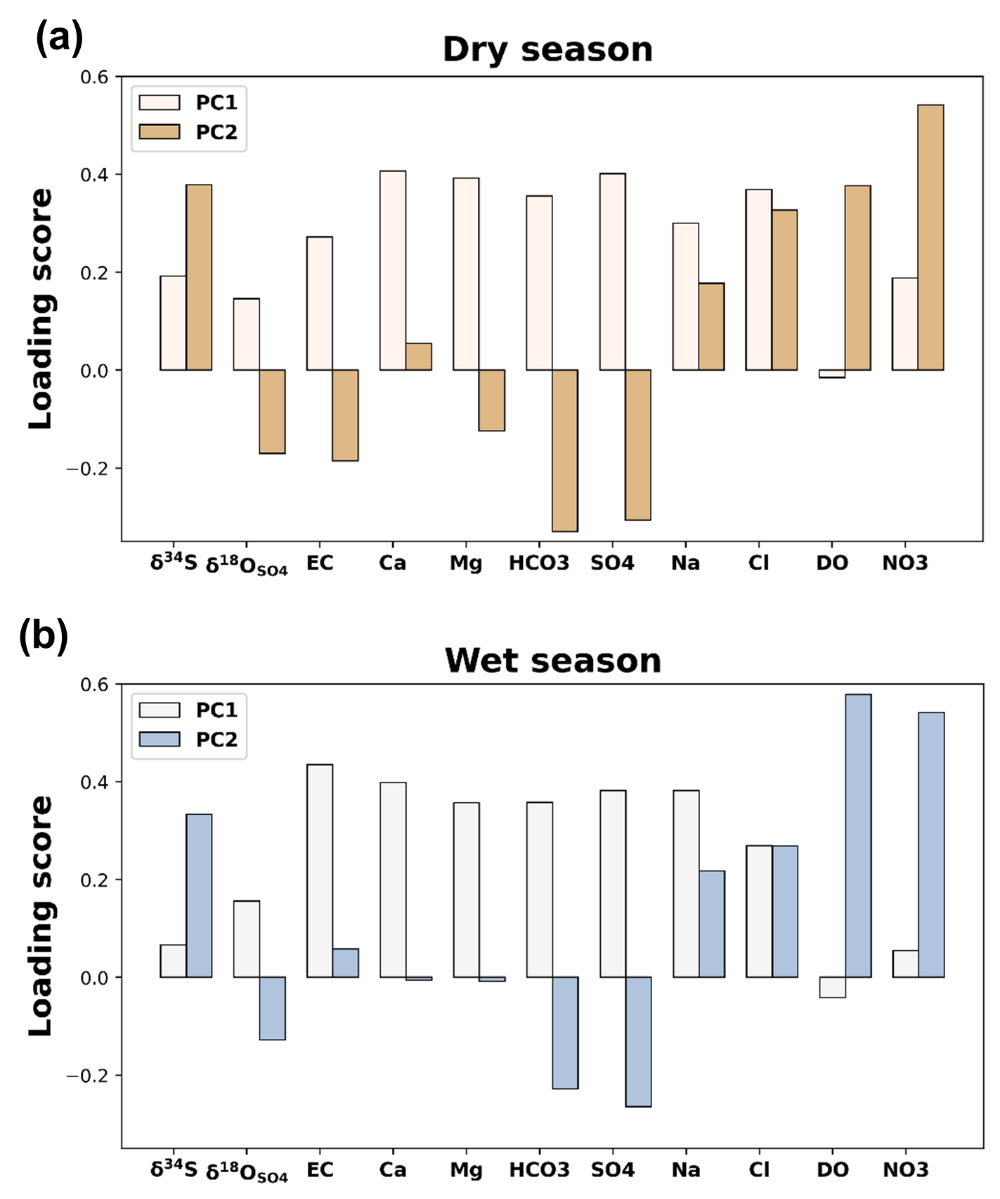
**

**Fig. S1 Bar graph of loadings in PCA showing the correlations between the input variables and PC1 and PC2 by dry and wet season.**

**Table S2 Loadings, eigen-values and variances of PCA input variables**

| **Variables** | **Dry season** | | **Wet season** | |
| --- | --- | --- | --- | --- |
|  | **PC1** | **PC2** | **PC1** | **PC2** |
| **δ^34^S_SO4_** | -0.19 | -0.38 | -0.07 | 0.33 |
| **δ^18^O_SO4_** | -0.15 | 0.17 | -0.16 | -0.13 |
| **EC** | -0.27 | 0.19 | -0.43 | 0.06 |
| **Ca^2+^** | -0.41 | -0.054 | -0.40 | -0.01 |
| **Mg^2+^** | -0.39 | 0.12 | -0.36 | -0.01 |
| **HCO_3_^-^** | -0.36 | 0.33 | -0.36 | -0.23 |
| **SO_4_^2-^** | -0.40 | 0.31 | -0.38 | -0.26 |
| **Na^+^** | -0.30 | -0.18 | -0.38 | 0.22 |
| **Cl^-^** | -0.37 | -0.33 | -0.27 | 0.27 |
| **DO** | 0.02 | -0.38 | 0.04 | 0.58 |
| **NO_3_^-^** | -0.19 | -0.54 | -0.05 | 0.54 |
| **Eigen value** | 0.38 | 0.17 | 0.45 | 0.16 |
| **Proportion (%)** | 45.29 | 20.70 | 51.96 | 18.41 |
| **Cumulative (%)** | 45.29 | 65.98 | 51.96 | 70.37 |

**Table S3** **Selected sulfur origins in groundwater as determined from published information**

| **Sources** | **δ^34^S_SO4_** | | **δ^18^O_SO4_** | | **Reference** |
| --- | --- | --- | --- | --- | --- |
|  | **Mean** | **SD** | **Mean** | **SD** |  |
| **Precipitation** | 4.87 | 1.38 | 14.57 | 1.55 | Yu and Park, 2004;  Lim et al. 2012 |
| **Sewage** | 9.93 | 4.05 | 10.68 | 2.29 | Bottrell et al. 2008;  Otero et al. 2008;  Shin et al. 2015 |
| **Soil** | 5.21 | 1.72 | 7.06 | 4.41 | Mayer et al. 1995;  Zhang et al. 2015 |
| **Sulfide**  **oxidation** | 1.96 | 2.23 | 0.82 | 5.49 | Park et al. 1991;  Jezierski et al. 2006;  Lipfert et al. 2007 |

Table S4 Proportion contribution result of Bayesian isotope mixing model by sources using δ^34^S_SO4_ and δ^18^O_SO4_

| **Input**  **variables** | **Unit** | | **Dry season (n=22)** | | | **Wet season (n=23)** | | |
| --- | --- | --- | --- | --- | --- | --- | --- | --- |
|  |  |  | **Group**  **D-1** | **Group**  **D-2** | **Group**  **D-3** | **Group**  **W-1** | **Group**  **W-2** | **Group**  **W-3** |
| **Precipitation** | | % | 12.78 | 8.85 | 18.15 | 8.30 | 3.17 | 8.90 |
| **Sewage** | |  | 12.48 | 7.15 | 5.02 | 4.66 | 4.34 | 2.45 |
| **Soil** | |  | 50.04 | 55.30 | 41.72 | 74.00 | 73.84 | 60.45 |
| **Sulfide**  **oxidation** | |  | 12.02 | 21.48 | 31.35 | 10.22 | 14.26 | 26.38 |
